# Case report: - A case report on the management of symptomatic Lingual throid

**DOI:** 10.1016/j.ijscr.2024.110005

**Published:** 2024-07-04

**Authors:** Hailemariam Kassaye Alebie, Yilkal Zemene Tasew, Fitusm Alemayehu Seyoum, Lense Gelaneh Negash

**Affiliations:** a Department of Otolaryngology-Head and Neck Surgery, St. Paul's Hospital Millennium Medical College, Addis Ababa, Ethiopia; b Department of Otolaryngology-Head and Neck Surgery, Head and Neck Surgeon, St. Paul's Hospital Millennium Medical College, Addis Ababa, Ethiopia

**Keywords:** Lingual thyroid, Ectopic thyroid tissue, Computed tomography

## Abstract

**Introduction:**

The lingual thyroid gland refers to ectopic thyroid tissue situated at the base of the tongue. This rare condition occurs when the thyroid gland fails to descend to its usual position in the pre-tracheal area during embryonic development.

**Case presentation:**

We present a case of a 23-year-old female who presented with throat discomfort and progressive difficulty of swallowing upon examination there was a mass at the level of the base of the tongue. She was investigated with a thyroid function test, neck ultrasound, at the tongue's base, and head and neck CT scan. With a diagnosed lingual thyroid she was managed initially with suppression therapy followed by elective surgical removal. The work has been reported in line with the SCARE criteria.

**Clinical discussion:**

The incidence of lingual thyroid is reported to be 1 in 100,000, with a higher prevalence among females, in a ratio of 3:1 compared to males. Symptoms can vary and may include difficulty swallowing (dysphagia), voice changes (dysphonia), upper airway obstruction, or occasional bleeding, and can manifest from infancy to adulthood.

**Conclusion:**

Lingual thyroid is a rare clinical anomaly treatment depending on the severity of symptoms, size of the lesion, sex and age of the patient, and thyroid function.

## Highlights

• The lingual thyroid gland refers to ectopic thyroid tissue situated at the base of the tongue.
• Symptoms can vary and can manifest from infancy to adulthood.
• A rare clinical anomaly treatment depends on the severity of symptoms.

## 1 Introduction

**Fig. 1:**
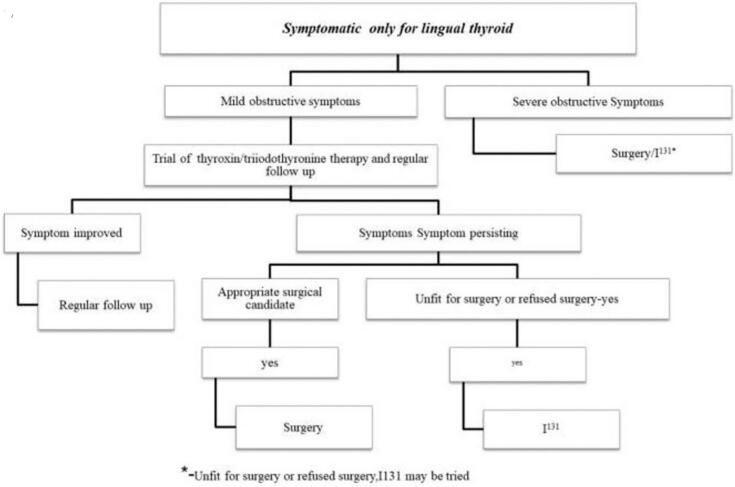
Management algorithm for symptomatic lingual thyroid [13].

Lingual thyroid is a rare medical condition characterized by the presence of thyroid tissue located in the midline at the base of the tongue. It arises during embryonic development when the thyroid gland fails to descend to its usual position [1,2]. The condition, known as ectopic thyroid tissue (ETT), is relatively uncommon, with an overall prevalence ranging from 1 in 100,000 to 300,000 individuals. Typically, ETT is situated in the region of the tongue's base, accounting for approximately 90 % of cases. Moreover, lingual thyroid tends to occur more frequently in females, comprising 70 % to 80 % of cases [3,4]. While many cases of lingual thyroid are asymptomatic, some individuals may experience local symptoms such as difficulty swallowing (dysphagia), changes in voice quality (dysphonia), obstruction of the upper airway, bleeding, and often hypothyroidism. Notably, clinical examination may not always reveal the presence of this lesion, despite its potential to cause mild to severe airway obstruction [[5], [6], [7]]. Diagnosis of lingual thyroid relies on the identification of thyroid tissue at the base of the tongue. Various imaging modalities, including ultrasound, CT scan, and nuclear medicine studies utilizing Technetium (Tc99m), can aid in evaluating the gland and confirming the diagnosis [9,10]. Additionally, it is clinically significant to examine the typical thyroid bed location radiologically, as in the majority of cases where a lingual thyroid is detected, it represents the sole functioning thyroid tissue in 70 % to 80 % of instances [8]. Once the diagnosis of lingual thyroid is established, the appropriate treatment strategy depends on factors such as the size of the gland, the nature of symptoms, thyroid function status, and histological findings [11,13]. Treatment options may include suppression therapy with levothyroxine, radioactive iodine ablation, or surgical removal of the lingual thyroid [7,12]. The recommended management algorithm for Lingual Thyroid depending on hormonal status of the patient and presence of symptoms specific to Lingual Thyroid (Fig. 1) [13].

## 2 Case report

**Fig. 2:**
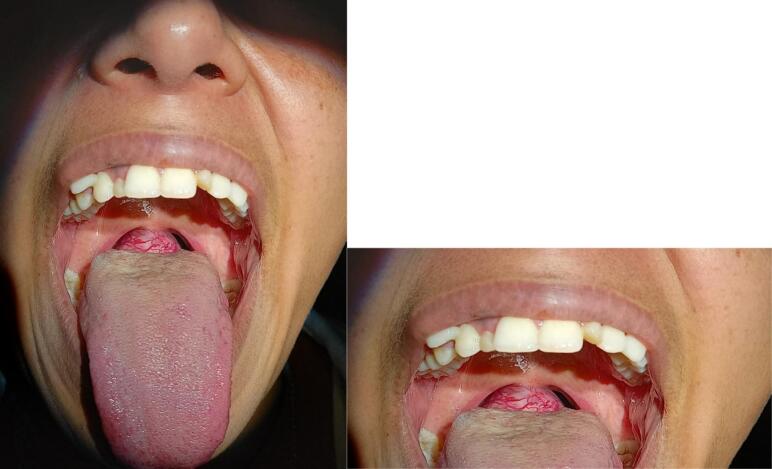
Preoperative examination revealed a mass that was covered with mucosa with numerous anastomosing blood vessels at the base of the tongue.

We present a case of a 23-year-old girl who presented with a complaint of throat discomfort and progressive dysphagia to solid foods for 11 months duration. Her past medical history was insignificant. On physical examination, it was noticed that she had a 3 cm × 2 cm midline, smooth, rubbery, and reddish mass at the base of the tongue. The surface of the swelling was smooth without any signs of ulceration, bleeding, or pus. However, numerous anastomosing blood vessels were seen over the mucosa of the swelling (Fig. 2). A neck examination revealed neither a palpable thyroid gland nor any other palpable masses.

**Fig. 3:**
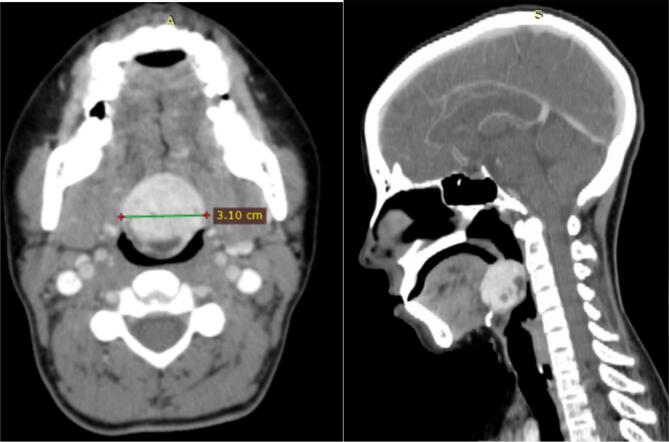
Post contrast standard Neck CT, there is a 3.10*3 cm mass hyperdense mass lesion at the level of the tongue base.

Serum thyroid stimulating hormone (TSH) was 0.94 mIU/L (Normal range: 0.350–5.000 mIU/L), with a normal free T4 level of 1.08 ng/dL (Normal range: 0.70–1.25 ng/dL). A neck ultrasound scan revealed the absence of a thyroid gland. Head and neck CT scan with contrast revealed a 3.1 cm × 3.0 cm × 1.2 cm homogeneously enhancing mass at the base of the tongue was noted, no thyroid tissue was visible on either side at the level of the thyroid cartilage (Fig. 3). Findings were compatible with ectopic lingual thyroid.

The final diagnosis was lingual thyroid. Suppression treatment with thyroid hormone was given for 28 days followed by elective surgical resection of the gland via transoral approach under general anesthesia induced via orotracheal intubation was carried out. The patient passed a smooth post-operative period, was discharged on the seventh day postoperatively on levothyroxine 5 μg/kg/day, was seen in the clinic in 1-month, 3-month, and 6-month intervals, and was found to be symptom-free.

## 3 Discussion

The thyroid gland emerges as the first endocrine gland during embryonic development. Initially, it manifests as a proliferation of endodermal cells originating from the foregut [1,2]. This process typically initiates around day 24 of development, with the cells commencing their migration from the foramen cecum situated at the posterior region of the tongue [1]. As they migrate caudally, they form the thyroglossal duct, eventually positioning themselves in the anterior neck, specifically between the 2nd and 5th tracheal rings [1,4]. However, in cases of ectopic thyroid tissue (ETT), there is a failure in the migration of the thyroid gland [4]. This failure is not limited to the path of the thyroglossal duct but can also involve locations in the mediastinum or other sub diaphragmatic areas. ETT, including its manifestation known as lingual thyroid when located at the base of the tongue, is relatively uncommon, with an overall prevalence estimated to range from 1 in 100,000 to 300,000 individuals [3]. Among ETT cases, lingual thyroid constitutes the majority (90 %), with a higher incidence in females (70 %–80 %) [3,6]. Lingual thyroid arises due to the failure of the thyroid anlage to descend from the foramen cecum of the tongue. Despite ongoing research, the precise reasons underlying this descent failure remain unknown [10]. Usually, the lingual thyroid is asymptomatic unless an increase in gland size occurs. In symptomatic cases, patients present with complaints of dysphagia, dysphonia, foreign body sensation in the throat, cough, pain, bleeding, and dyspnea [2,4]. Hypothyroidism in up to 70 % of patients [2]. A study looking at 200 autopsies found, that 10 % of individuals had thyroid tissue ranging from microscopic to 1 cm, indicating how common the condition could be [11]. Intuitively, ectopic thyroid gland tissue will be indistinguishable from orthotopic thyroid gland tissue on imaging, although differently shaped [1]. On physical examination. it presents as a midline nodular mass at the base of the tongue. On video laryngoscopy examination, the lingual thyroid appears as a smooth surface mucosal lesion with vascularity [4,5]. An essential part of the examination is palpation of the neck to check the presence or absence of the thyroid gland in a normal position [5].

Investigations for the diagnosis and treatment plan for lingual thyroid include radionuclide, Technetium-99 m, and iodine-123 thyroid scans in addition to serum thyroid profiles (T3, T4, and TSH) (1). The most useful method to localize the lingual thyroid is thyroid scintigraphy with 123I‑iodine or 99mTctechnetium, which shows the uptake of radionuclide activity at the tongue base and no activity in the normal location in the neck (3). Ultrasonography of the neck is a noninvasive and readily available tool in the evaluation of the presence or absence of thyroid tissue in normal locations and conducting initial detection of the ETT (8). CT scan and MRI are also helpful to define the location and to evaluate the characteristics of ETT [2,3]. Our case did not reveal any normal thyroid gland on neck ultrasonography and CT scan examination. FNAC can not only help to confirm the diagnosis of ETT but also to rule out the potential of malignancy [3]. Technetium (Tc99m) thyroid scintigraphy may avoid the need for a diagnostic biopsy, which involves the risk of bleeding or acute thyrotoxicosis [4].

Treatment depends on the severity of symptoms, size of the lesion, sex and age of the patient, and thyroid function [5,6]. No treatment is required when the lingual thyroid is asymptomatic [2]. Conservative treatment with substitutive hormone in patients with mild symptoms [2]. Suppressive therapy with thyroid hormone should be tried first to decrease the size of the gland. Indications for surgical removal are malignancy, cystic degeneration, bleeding, ulceration, uncontrolled hyperthyroidism, and severe compressive, and respiratory symptoms [4]. Alternatives to surgery are embolization alone or transoral laser excision, radio frequency, or radioiodine previous to surgery. The transoral approach offers the best approach among different types of surgical access and provides good exposure, better postoperative recovery, and is less traumatic for the patient [2,4,6]. In our patient elective surgery following the suppression therapy was done. Additionally, levothyroxine therapy was initiated after surgical excision as the lingual thyroid is the only functioning thyroid tissue.

## 4 Conclusion

Lingual thyroid is a rare clinical anomaly representing faulty migration of the normal thyroid gland that needs careful diagnostic workup including clinical examination, biochemical tests, and imaging methods to plan the management. Treatment depends on the severity of symptoms, size of the lesion, sex and age of the patient, and thyroid function. Lingual thyroid with hypothyroidism and no neck regional symptoms can be conservatively treated, however, regular follow-up is required for the prevention of the potential risk of malignant transformation.

## Informed consent

Written informed consent was obtained from the patients to publish this case report and accompanying images. A copy of the written consent is available for review by the Editor-in-Chief of this journal on request.

## Ethical approval

Our institution does not require ethical approval for reporting individual cases (We maintained a high level of respect for anonymity and confidentiality when presenting the patient in our case series.)

## Funding

This research received no specific grant from funding agencies in the public, commercial, or not-for-profit sectors.

## Author contribution

Hailemariam Kassaye Alebie: Writing the paper, Corresponding author and Author Guarantor.

Yilkal Zemene Tasew: Study concept, and paper edition.

Fitusm Alemayehu Seyoum: Paper edition.

Lense Gelaneh Negash: Paper edition.

## Guarantor

Hailemariam Kassaye Alebie, Department of Otolaryngology-Head and Neck Surgery, St. Paul’s Hospital Millennium Medical College, Addis Ababa, Ethiopia

## Research registration number

On processes.

## Declaration of competing interest

All authors declare no conflicts of interest in this article.

## Data Availability

All data are included within the article.
